# Verification of the accuracy of a photon dose‐calculation algorithm

**DOI:** 10.1120/jacmp.v3i1.2589

**Published:** 2002-01-01

**Authors:** Kent A. Gifford, David S. Followill, H. Helen Liu, George Starkschall

**Affiliations:** ^1^ Department of Radiation Physics The University of Texas M. D. Anderson Cancer Center 1515 Holcombe Boulevard Houston Texas 77030

**Keywords:** photon‐dose calculations, radiation treatment planning, quality assurance, algorithm verification

## Abstract

An extensive set of measured data was developed for the purpose of verifying the accuracy of a photon dose‐calculation algorithm. Dose distributions from a linear accelerator were measured using an ion chamber in a water phantom and thermoluminescent dosimeters in a heterogeneous anthropomorphic phantom. Test cases included square fields, rectangular fields, fields having different source‐to‐surface distances, wedged fields, irregular fields, obliquely incident fields, asymmetrically collimated fields with wedges, multileaf collimator‐shaped fields, and two heterogeneous density cases. The data set was used to validate the photon dose‐calculation algorithm in a commercial radiation treatment planning system. The treatment planning system calculated photon doses to within the American College of Medical Physics (AAPM) Task Group 53 (TG‐53) criteria for 99% of points in the buildup region, 90% of points in the inner region, 88% of points in the outer region, and 93% of points in the penumbra. For the heterogeneous phantoms, calculations agreed with actual measurements to within ±3%. The monitor unit tests revealed that the 18‐MV open square fields, oblique incidence, oblique incidence with wedge, and mantle field test cases did not meet the TG‐53 criteria but were within ±2.5% of measurements. It was concluded that (i) the photon dose calculation algorithm used by the treatment planning system did not meet the TG‐53 criteria 100% of the time; (ii) some of the TG‐53 criteria may need to be modified, and (iii) the generally stated goal of accuracy in dose delivery of within 5% cannot be met in all situations using this beam model in the treatment planning system.

PACS number(s): 87.53.–j, 87.66.–a

## INTRODUCTION

Before implementing a radiation treatment planning system in the clinic, the dose‐calculation algorithm must be validated using rigorous, clinically relevant criteria. The algorithm must accurately calculate dose distributions for a variety of clinical beam configurations. Verifying the accuracy of the dose computation requires a comprehensive set of test cases. Although dose‐calculation algorithms can generally calculate dose distributions for a radiation beam at normal incidence on a water phantom, their accuracy in a variety of clinical situations may be questionable. Some such situations include oblique incidence of the radiation beam (e.g., tangential breast irradiation) or multiple density heterogeneities (e.g., lung irradiation).

The American Association of Physicists in Medicine (AAPM) Radiation Therapy Committee Task Group 23 (TG‐23)^1^ developed a test package for verifying the accuracy of photon‐beam dose‐calculation algorithms. Data for the test cases were acquired for two beam energies from two clinical linear accelerators: a 4‐MV *x*‐ray beam from a Clinac‐4 (Varian Oncology Systems, Palo Alto, CA), and an 18‐MV *x*‐ray beam from a Therac‐20 (Atomic Energy of Canada, Ltd., Kanata, Ontario, Canada). Although TG‐23 used 13 test cases for algorithm verification, several clinically significant situations were not included. For example, the TG‐23 cases were developed at a time when three‐dimensional (3D) radiation treatment planning was just beginning; consequently, TG‐23 did not include test cases that examined issues present in 3D treatment planning such as 3D density heterogeneities. Inclusion of 3D test cases is essential because the patient cannot always be modeled as a two‐dimensional (2D) object. In fact, many clinics no longer use 2D treatment planning. Additionally, when treating the thoracic region, some beams have to traverse bone and then lung tissue. The differences in material composition in this region can significantly affect dose‐calculation algorithms because electronic equilibrium is not established at the interfaces. The representation of inhomogeneous media in the TG‐23 cases is only two‐dimensional.

Another shortcoming of the TG‐23 report is that the wedged‐field case presented therein was that of a 45° wedge. Although this is a clinically valid situation, we have found that a 60° wedge has been more difficult to model than a 45° wedge.^2^ Moreover, the wedged beam is presented at normal incidence on the phantom, while a common clinical use of wedged beams is at an oblique incidence. The TG‐23 report also presented a significant example of an irregular field: the “hockey stick” field. Another clinically relevant but more complex irregular field is the mantle field used to treat Hodgkin's disease. Although TG‐23 covered oblique incidence, the clinical range of obliquities was not explored. The range of obliquities commonly encountered in current radiation therapy includes obliquities greater than 45°, such as the angles encountered when treating breast cancer using tangential fields. Additionally, asymmetric collimation was not addressed. Finally, the TG‐23 data set did not include absolute dose determination, which could be used for monitor unit (MU) verification. A more recent study done has extended the TG‐23 data set to include some of these additional capabilities.^3^

Another group that addressed algorithm verification was the Electron Collaborative Working Group (ECWG).^4^ The ECWG's experiments were designed to test the fundamental characteristics of electron dose‐calculation algorithms, as well as the accuracy of these algorithms in clinical situations. Measurements were performed for situations including variation of energy, source‐to‐skin distance (SSD), electron applicator, field shaping, and irregular surfaces and heterogeneities (1D, 2D, and 3D) using air, lung, and bone substitutes. A useful outcome from the ECWG data was that their data were formatted and made available for distribution so that other institutions could apply them to verify their electron dose‐calculation algorithms. The ECWG dataset was recently reviewed and expanded by Boyd *et al.* ^5^

Given that several clinically significant situations were not included in the 13 TG‐23 test cases, the AAPM Radiation Therapy Committee Task Group 53 (TG‐53) report^6^ suggested several photon dose‐calculation verification situations. The test cases generated in the present study as well as the methodology used in generating these test cases evolved from the TG‐23 and TG‐53 work.

The purpose of this study is to generate a data set that could be used for evaluating photon dose‐calculation algorithms used in contemporary treatment planning systems. To achieve this goal, several revisions were made to the data set described in the TG‐23 project. First, the test cases pertaining to inhomogeneous media were 3D instead of 2D. Additional test cases were needed; these included oblique incidence with a wedged field, significant asymmetric (half beam) collimation, a mantle field, a field defined using multileaf collimators (MLCs), a 3D representation of the lung with a tissue‐bone interface, and a neck phantom with a tissue‐air interface. The accuracy of treatment planning system MU calculations was also assessed.^7^

Test cases representing 12 different clinical setups were included in the data set to verify the accuracy of the photon dose‐calculation algorithm. These setups included open square and rectangular fields, extended SSDs, wedged fields, irregular fields, short SSDs, oblique incidence, as well as the cases described in the previous paragraph. Also, the data set contained measured data, including fractional depth dose (FDD) curves, sagittal and transverse beam dose profiles, total scatter factors, and point doses in the heterogeneous case.

This data set was then applied to a commonly used photon‐beam dose‐calculation algorithm with goals of (i) validating the determinations of the parameters used in the beam model, and (ii) evaluating the accuracy of the dose calculated by the model in various clinically relevant situations.

The data set was developed specifically for photon dose‐calculation verification. Data from the open square field test case can be used to generate a beam model in the treatment planning system. The other test cases can then be used to verify the photon dose‐calculation algorithm under the particular clinical configurations. It is our intention that the data set developed in this work be appropriately formatted for distribution in the same manner as the ECWG data set.

## MATERIALS AND METHODS

### A. Measurement of the data set

In all of the test cases, with the exception of the heterogeneity cases, the FDD curves and profiles in the *x* and *y* directions were measured in a water phantom (Wellhöffer, Schwarzenbruck, Germany). Unless otherwise noted, the profile depths were 1.2, 4.0, 10.0, and 20.0 cm for 6‐MV beams, and 3.2, 6.0, 10.0, and 20.0 cm for 18‐MV beams. These depths were chosen so that the majority of the calculated profiles would not have to be interpolated from the calculated dose matrix. In each case, the shallowest depth was selected so that the depth would be in the buildup region if the field were small, and close to $d_{\max}$ if the field were large.

Data were acquired using a Clinac 2100C linear accelerator (Varian Medical Systems, Inc., Palo Alto, CA). Two $0.1‐{\;\text{cm}}^{3}$ ion chambers (Model No. N23323, PTW‐Freiburg, Freiburg, Germany) were employed to acquire data in the water phantom. Off‐axis profiles not passing through the central ray of the beam were measured at a distance equal to 80% of the half‐width of the radiation field at the particular depth and in the positive direction (coordinate system discussed below). For example, the off‐axis profiles measured for the 5cm×5cm open field test case at a depth of 10 cm were +2.2cm away from the central axis. While the actual location of the off‐axis profile is somewhat arbitrary, selection of this location places the profile in a region in which the beam is still reasonably flat, but may exhibit significant differences from a profile passing through the central axis of the beam. Verification of MU calculations was also performed.

Thermoluminescent dosimeters (TLDs) were used to measure the absolute dose in the heterogeneous phantoms. Each TLD consisted of approximately 25 mg of TLD‐100 powder (Harshaw Chemical Co., Solon, OH). The cylindrical active volume of the detector was 2‐mm diameter by 3‐mm length. To calibrate the TLD, a set of reference TLDs was irradiated at a specified reference dose at the depth of the maximum dose in a 10cm×10cm field for each energy. Three readings were obtained at each measurement point.

The coordinate system for this study was defined as follows: The origin was located at the intersection of the central axis of the beam with the surface of the phantom. For eight of the ten cases, the origin was the machine isocenter. Facing the gantry, the *x* axis pointed to the observer's right, the *z* axis pointed upward, and the *y* axis was chosen so that the coordinate system would be right‐handed. These coordinates were consistent with the specifications in the International Electrotechnical Commission 61217 document.^8^

Unless otherwise noted, all profiles in the water phantom were normalized to the FDD at a depth of 10 cm for the particular field size and clinical setup. Consequently, the central axis FDD at a depth of 10 cm was equal to 1.00. It was necessary to select a point for normalization in order that absolute comparisons be made, and the selection of the depth at 10 cm was somewhat arbitrary. Total scatter factors were then referenced to an ion‐chamber reading at a depth of 10 cm for a 10cm×10cm field.

### B. Dose‐calculation algorithm

The photon dose‐calculation algorithm evaluated in this study is the convolution/superposition algorithm that was introduced by Mackie *et al* ^9^ and extended by Papanikolaou *et al.* ^10^ to polyenergetic spectra. The implementation of the dose‐calculation algorithm in the particular commercial treatment planning system (Pinnacle^3^; ADAC Laboratories, Milpitas, CA) has been described previously.^2^

The set of beam model parameters used in the clinic was employed in all open field test cases. However, the wedge models had to be commissioned specifically for this study because the clinical model uses dynamic wedges, while the measured test cases use physical wedges. (At the time this study was initiated, the version of the treatment planning system did not support dose calculations using dynamic wedges.) Consequently, parameters appropriate to the physical wedges had to be determined. These parameters were obtained using guidelines recommended by Starkschall *et al.* ^2^ Table I displays the beam parameters for the 18‐MV open field models.

**Table I:** Open field beam parameters for the 18‐MV model. Numbers in roman font are modeled values, while numbers in italic are interpolated values.

Field size (cm^2^)														
	4	5.2	6.2	8	10.2	12.2	15.2	18.2	20.4	22.2	25.4	30.2	35.2	40.4
Spectrum (MeV)														
0.10	0.017	0.017	0.017	0.017	0.017	0.017	0.077	0.017	0.017	0.017	0.017	0.017	0.017	0.017
0.20	0.031	0.031	0.031	0.031	0.031	0.031	0.031	0.031	0.031	0.031	0.031	0.031	0.031	0.031
0.30	0.045	0.045	0.045	0.045	0.045	0.045	0.045	0.045	0.045	0.045	0.045	0.045	0.045	0.045
0.40	0.057	0.057	0.057	0.057	0.057	0.057	0.057	0.057	0.057	0.057	0.057	0.057	0.057	0.057
0.50	0.069	0.069	0.069	0.069	0.069	0.069	0.069	0.069	0.069	0.069	0.069	0.069	0.069	0.069
0.60	0.080	0.080	0.080	0.080	0.080	0.080	0.080	0.080	0.080	0.080	0.080	0.080	0.080	0.080
0.80	0.101	0.101	0.101	0.101	0.101	0.101	0.101	0.101	0.101	0.101	0.101	0.101	0.101	0.101
1.00	0.118	0.118	0.118	0.118	0.118	0.118	0.118	0.118	0.118	0.118	0.118	0.118	0.118	0.118
1.25	0.137	0.137	0.137	0.137	0.137	0.137	0.137	0.137	0.137	0.137	0.137	0.137	0.137	0.137
1.50	0.152	0.152	0.152	0.152	0.152	0.152	0.152	0.152	0.152	0.152	0.152	0.152	0.152	0.152
2.00	0.173	0.173	0.173	0.173	0.173	0.173	0.173	0.173	0.173	0.173	0.173	0.173	0.173	0.173
3.00	0.190	0.190	0.190	0.190	0.190	0.190	0.190	0.190	0.190	0.190	0.190	0.190	0.190	0.190
4.00	0.184	0.184	0.184	0.184	0.184	0.184	0.184	0.184	0.184	0.184	0.184	0.184	0.184	0.184
5.00	0.165	0.165	0.165	0.165	0.165	0.165	0.165	0.165	0.165	0.165	0.165	0.165	0.165	0.165
6.00	0.141	0.141	0.141	0.141	0.141	0.141	0.141	0.141	0.141	0.141	0.141	0.141	0.141	0.141
8.00	0.093	0.093	0.093	0.093	0.093	0.091	0.089	0.087	0.085	0.084	0.083	0.080	0.078	0.075
10.00	0.065	0.063	0.061	0.058	0.054	0.053	0.050	0.047	0.040	0.045	0.044	0.043	0.041	0.040
15.00	0.017	0.015	0.014	0.012	0.009	0.008	0.007	0.005	0.004	0.004	0.003	0.003	0.003	0.002
20.00	0.001	0.001	0.001	0.000	0.000	0.000	0.000	0.000	0.000	0.000	0.000	0.000	0.000	0.000
Incident fluence														
Fluence increase/cm	0.0060	0.0060	0.0060	0.0060	0.0060	0.0060	0.0060	0.0060	0.0060	0.0060	0.0061	0.0062	0.0064	0.0065
Cone radius	7.0	7.0	7.0	7.0	7.0	7.0	7.0	7.0	7.0	8.0	10.0	13.0	17.0	20.0
Source *x*	0.08	0.08	0.08	0.08	0.08	0.08	0.08	0.08	0.08	0.08	0.08	0.08	0.08	0.08
Source *y*	0.08	0.08	0.08	0.08	0.08	0.08	0.08	0.08	0.08	0.08	0.08	0.08	0.08	0.08
Gaussian height	0.05	0.05	0.05	0.05	0.05	0.05	0.05	0.05	0.05	0.05	0.04	0.04	0.03	0.02
Gaussian width	1.0	1.0	1.0	1.0	1.0	1.0	1.0	1.0	1.0	1.0	1.0	1.0	1.0	1.0
Jaw transmission	0.01	0.01	0.01	0.01	0.01	0.01	0.01	0.02	0.02	0.02	0.02	0.03	0.03	0.04
Modifiers														
Modifier scatter factor	0.0	0.0	0.0	0.0	0.0	0.0	0.0	0.0	0.0.	0.0	0.0	0.0	0.0	0.0
Electron contamination														
Maximum depth	6	6	6	6	6	6	6	6	6	6	6	6	6	6
Surface dose	0.600	0.500	0.500	0.400	0.300	0.300	0.300	0.300	0.300	0.297	0.293	0.285	0.278	0.270
Depth coefficient	1.5	1.4	1.3	1.2	1.0	1.0	1.0	1.0	1.0	1.0	1.0	1.0	1.0	1.0
Off‐axis coefficient	0	0	0	0	0	0	0	0	0	0	0	0	0	0
DF^a^	0	0	0	0	0	0	0	0	0	0	0	0	0	0
SF^a^	1	1	1	1	1	1	1	1	1	1	1	1	1	1
C1^a^	0.001	0.001	0.001	0.001	0.001	0.001	0.001	0.001	0.001	0.001	0.001	0.001	0.001	0.001
C2^a^	0.9	0.9	0.9	0.9	0.9	0.9	0.9	0.9	0.9	0.9	0.9	0.9	0.9	0.9
C3^a^	0.1	0.1	0.1	0.1	0.1	0.1	0.1	0.1	0.1	0.1	0.1	0.1	0.1	0.1
Spectral factors														
Off‐axis softening	0	0	0	0	0	0	0	0	0	0	0	0	0	0
Modeling geometry														
Grid resolution	0.4	0.4	0.4	0.4	0.4	0.4	0.4	0.4	0.4	0.4	0.4	0.4	0.4	0.4
Phantom size‐lateral	50	50	50	50	50	50	50	50	50	52	55	60	65	70
Phantom size‐depth	50	50	50	50	50	50	50	50	50	50	50	50	50	50

a DF, SF, CI, C2, C3 are parameters used to model electron contamination. Values of DF and SF used here indicate that these parameters are not used on the model; CI, C2, and C3 model the field size‐dependence of the electron contamination.

All dose calculations were performed on version 4.2 of the treatment planning system. A 4‐mm dose grid was used in each test case because this is the grid typically used for calculations in this institution. With the exception of the oblique test cases, all test cases were calculated using the water phantom option provided in the treatment planning system. This option replaces the actual CT data set with a unit density data set and a constant SSD equal to the SSD along the central axis of the beam. Doses in the oblique test cases and the anthropomorphic phantom test cases were computed with the heterogeneity correction option in the treatment planning system, which used the actual CT data set along with a table that converted CT voxel values to electron densities.

### C. Data comparison and presentation

After dose computation, files containing dose matrices were stripped of unwanted text and formatted so that they could be imported into a commercial image‐manipulation and data‐analysis software system (IDL, Research Systems Inc., Boulder, CO). Profiles were overlaid to compare the computed and measured data. The maximum deviation in each part of the radiation field was tabulated for each test case. Monitor unit comparisons were included in another table.

The report on quality assurance of radiation treatment planning produced by the AAPM TG‐53^6^ specified acceptance criteria in terms of the percent difference and distance difference. Consequently, these criteria were used for comparing the data in this study. Percent differences were calculated as the difference between the dose calculated at a data point and the dose measured at the same point multiplied by 100%. Because the measured and calculated data were normalized to the same value, no reference value for the percent difference was needed. To calculate the distance differences in high‐dose‐gradient regions (primarily beam penumbra), we identified the measured data points that bracket the calculated data point value. The coordinate of the point with the same dose as that of the calculated point was interpolated between these two measured data point coordinates. The distance between the calculated and measured points was calculated. Only those calculated points lying within the boundaries established by the TG‐53 definition of penumbra, that is, points within 0.5 cm from the edge of the beam or beam modifier, were tested as penumbra points.

Because of the enormity of the data set, data analysis and presentation methods were used that minimized the number of plots while allowing appropriate analysis of the data. Profile overlays, which are plots of the calculated and measured profile on the same axis, identified regions of discrepancy. In regions of a high‐dose gradient, a percent difference between calculated and measured dose values is not clinically significant. In this region, the distance difference was utilized.

## TEST CASES

### A. Test case 1: Water phantom, 100‐cm SSD, open square field

**Figure 1:**
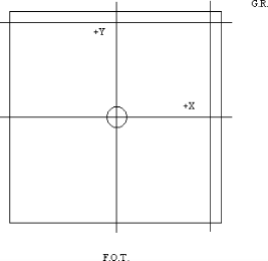
Beam's eye view of the field orientation for all open square fields with normal incidence. The profiles are marked by lines that extend out of the field, and the central axis is identified by the circle at the center of the field. G.R.; gantry right, F.O.T.; foot of table.

This case tested the ability of the photon dose‐calculation algorithm to reproduce the dose distribution in a configuration similar to the configuration used to measure the original input data. Data were obtained for the 6‐ and 18‐MV photon beams with fields of 5cm×5cm and 25cm×25cm. Four profiles were measured for each depth, two passing through the central axis and two passing through a specified off‐axis position. Figure 1 illustrates the beam's‐eye‐view (BEV) orientation and locations of the profiles measured for all open square fields.

### B. Test case 2: Water phantom, extended SSD (125 cm), open square field

The extended SSD setup tested the ability of the treatment planning system to predict the increase in penumbra width and change in depth dose due to the increased distance from the source. Data were obtained for the 6‐ and 18‐MV beams using 8cm×8cm and 20cm×20cm fields at an SSD of 125 cm.

### C. Test case 3: Water phantom, 100‐cm SSD, open rectangular field

**Figure 2:**
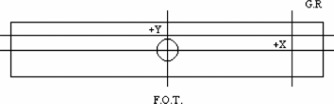
Locations of the profiles for the 25cm×5cm field in test case 3. The beam's eye view for the 5cm×25cm field is the figure rotated 90°, while gantry right and foot of table remain in the same position. The central axis is marked by the circle. G.R.; gantry right, F.O.T.; foot of table.

This case tested the ability of the dose‐calculation engine to compute the dose in an elongated rectangular field based on data input from square fields. The dose distributions in rectangular fields of 5cm×25cm and 25cm×5cm were measured for the 6‐ and 18‐MV beams at an SSD of 100 cm. Figure 2 illustrates the BEV locations of the profiles for this case.

### D. Test case 4: Water phantom, 100‐cm SSD, wedged square fields

This case tested the ability of the algorithm to reproduce dose distributions in wedged fields. The wedge angles used were 45° and 60°. Each wedge was oriented in the *x* direction with the thin end pointing to gantry right. The 45° and 60° wedges were chosen because they were the most difficult to model as their thickness maximized beam hardening and wedge scatter. These data were acquired at an SSD of 100 cm for the 6‐ and 18‐MV beams at field sizes of 6cm×6cm and 20cm×20cm for the 45° wedge, and 15cm×15cm for the 60° wedge.

### E. Test case 5: Water phantom, 100‐cm SSD, mantle field

**Figure 3:**
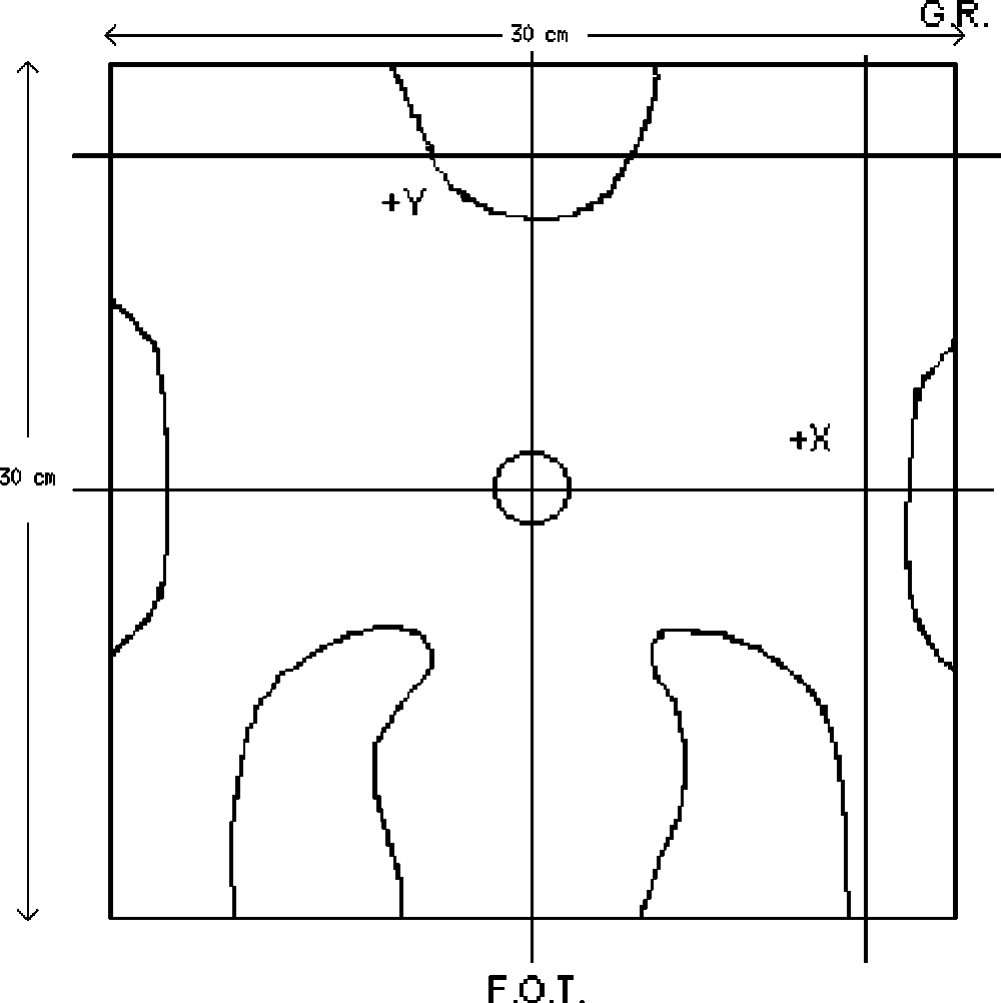
Locations of the profiles for test case 5. The off‐axis profiles that did not pass through the midline of the radiation field were normalized using the upper right‐hand corner off‐axis profile intersection point. The central axis is marked by the circle. G.R.; gantry right, F.O.T.; foot of table.

To treat Hodgkin's lymphoma and certain other cancers, very irregularly shaped fields are used to conform the beam to the target site and spare critical structures. A mantle field, which is used in the treatment of Hodgkin's lymphoma, was used to test the algorithm's accuracy for irregularly shaped fields. Data were obtained at an SSD of 100 cm for the 6‐ and 18‐MV beams with a collimator setting of 30cm×30cm. All data were normalized to the FDD at a depth of 10 cm from the surface of the water phantom with the mantle field block in place. Figure 3 illustrates the locations of the profiles.

### F. Test case 6: Water phantom, open square field, isocentric setup

Although beam data are normally acquired at a fixed SSD of 100 cm, most patients are treated isocentrically, using SSDs that are less than the source‐to‐axis distance (SAD). Because of this, the dose‐calculation algorithm was tested using an isocentric setup. Dose distributions for a 6‐MV beam were measured at an SSD of 90 cm with an 11.1cm×11.1cm collimator setting, while

18‐MV dose distributions for an 18‐MV beam were measured at an SSD of 80 cm with a 12.5cm×12.5cm collimator setting. These configurations corresponded to isocenter depths of 10 and 20 cm, respectively.

### G. Test case 7: Water phantom, 100‐cm SSD, open square field, oblique incidence

**Figure 4:**
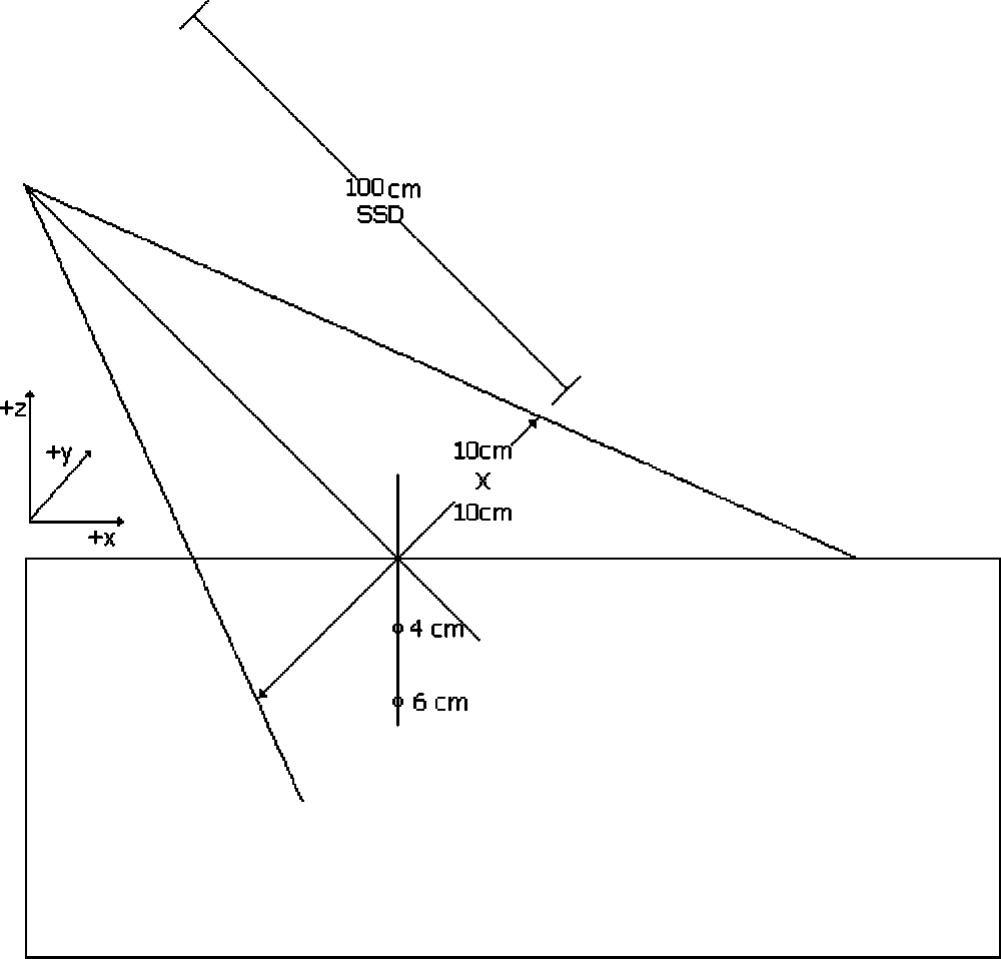
Side view of the oblique‐incidence irradiation geometry. The normalization points for the 305° and 330° gantry angles are 4 and 6 cm, respectively, along a normal surface of the water phantom.

This case tested the ability of the algorithm to calculate the dose for an obliquely incident beam. In general, dose distributions for oblique incidence should differ from those for normal incidence because an obliquely incident beam causes different amounts of scatter from different parts of the phantom than does a normally incident beam.^11^ For this test, the gantry angles were 330° and 305° for each energy in a 10cm×10cm field at an SSD of 100 cm. All profiles were measured either perpendicular or parallel to the surface. In addition, data for the 305° gantry angle were normalized to the FDD at a depth of 4 cm from the surface of the phantom for the particular field size and energy, while those for the 330° gantry angle were normalized to the FDD at a depth of 6 cm. Figure 4 illustrates a side view of the oblique incidence setup.

### H. Test case 8: Water phantom, 100‐cm SSD, asymmetric jaws (half beam and 45° wedge)

**Figure 5:**
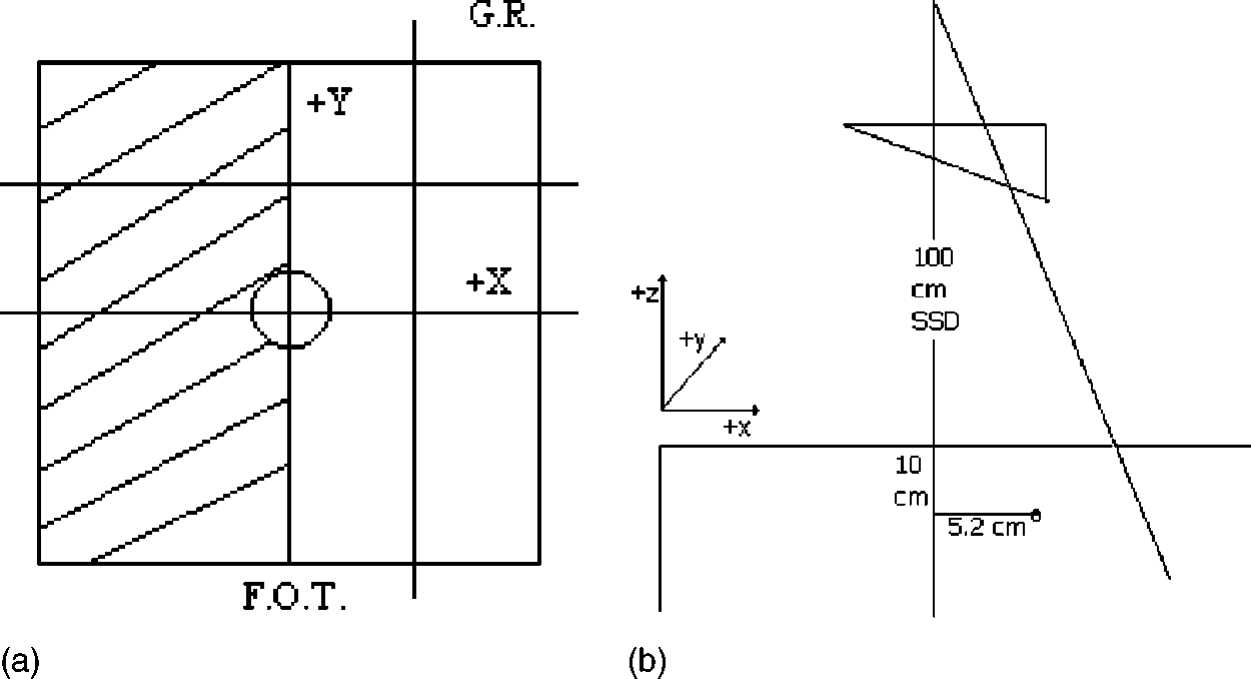
(a) Locations of the profiles for test case 8. The profiles are marked by lines that extend out of the edge of the field. The central axis is marked by the circle. G.R.; gantry right, F.O.T.; foot of table. (b) The side view of the asymmetrically collimated (half beam), wedged‐field setup. The normalization point is 10.0 cm from the surface and 5.2 cm away from the central axis.

This case, which was not included in previous test sets, is another test of the ability of the dose‐calculation algorithm to produce an accurate dose distribution using a nonstandard, although common, beam configuration. Photon‐beam dose distributions of 6 and 18 MV at and SSD of 100 cm were measured with an asymmetrically collimated 10cm×20cm field and a 45° wedge. The wedge was oriented in the *x* direction with the toe pointing to gantry left, and the FDD was measured beginning at a point +5.2cm in the *x* direction. All of the data were normalized to the FDD at a depth of 10 cm from the surface of the phantom and +5.2cm away from the central axis in the *x* direction for the particular energy. (Figure 5a) illustrates the locations of the profiles for this case, while (Fig. 5b) illustrates the side view of the irradiation setup.

### I. Test case 9: Water phantom, 100‐cm SSD, wedged field, oblique incidence

In the irradiation of certain sites, for example, breast and vocal cords, oblique incidence is compensated for by the use of wedges. In this case, a 45° wedge was implemented with a gantry angle of 315°. The wedge was oriented in the *x* direction with the toe pointing to gantry left, and 10cm×10cm field was used at each energy. All of the data were normalized to the FDD at a depth of 5 cm from the surface of the phantom for the particular field size and energy. All profiles were measured either perpendicular or parallel to the surface.

### J. Test case 10: Water phantom, 100‐cm SSD, MLC field

**Figure 6:**
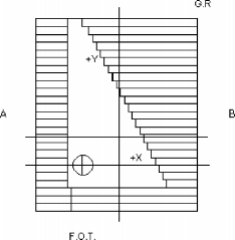
Locations of the profiles for test case 10. The central axis is marked by the circle G.R.; gantry right, F.O.T.; foot of table.

Dose‐calculation algorithms generally make approximations in modeling the leaves of a multileaf collimator (MLC). For example, they may not model interleaf leakage or the rounded leaf edges. This case tested the ability of the photon dose‐calculation algorithm to predict the dose

under MLC leaves and the leaf leakage through them. Measurements for an 80‐leaf Varian MLC were obtained using the following collimater settings: x1=6.0,y1=5.0,x2=12.0, and y2=19.2. The shape of the field was a right triangle, with the hypotenuse at gantry right. Figure 6 illustrates the locations of the profiles for this case.

### K. Test case 11: Heterogeneous medium (lung phantom)

**Figure 7:**
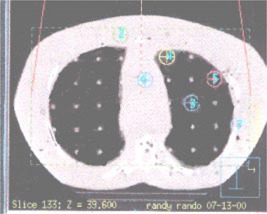
(Color) Locations of the test points within the anthropomorphic phantom for the lung test case.

In treating lung cancer, the radiation beam has to travel through soft tissue, bone (ribs), and lung tissue. In this case, measurements were made in a heterogeneous anthropomorphic phantom (Rando® phantom; Radiology Support Devices, Inc., Long Beach, CA) to simulate the lung configuration. An 18‐MV beam was employed with a 26cm×12cm field, and 500 MU were delivered at an SSD of 100 cm. External spot markers (Beekley Corp., Bristol, CT) were placed on the phantom surface to allow accurate, reproducible positioning. Also, measurements were taken using TLD‐100 powder at specific points in the lungs; the powder was encapsulated in polyethylene plugs that fit into predrilled holes in the phantom. The TLD reader (Harshaw Chemical Co.) was used to measure the absorbed dose for each TLD measurement. At the end of the measurement session, three TLD standards were irradiated to 520 MU in a water phantom under calibration conditions (100‐cm SSD, 10cm×10cm field, $d_{\max}$) to provide a reference dose of 520 cGy for comparison with phantom measurements. Appropriate corrections for TLD energy dependence, fading and nonlinearity (nonlinearity of TLD defined for doses between 0 and 600 cGy) were applied to all TLD readings. Three sets of TLD measurements were performed to assess the precision of measurement. Figure 7 shows the locations of the measurement points within the phantom.

### L. Test case 12: Heterogeneous medium (neck phantom)

**Figure 8:**
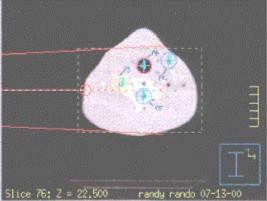
(Color) Locations of the test points within the anthropomorphic phantom for the neck test case.

To treat certain head and neck cancers, the radiation beam must pass consecutively through tissue, bone, and air, with a nonequilibrium condition present in the bone‐air region. In this case, neck treatment was simulated using the Rando® phantom. A 10cm×14cm field was used with a 6‐MV beam, and 500 MU were delivered at an SSD of 100 cm. The reference dose for this test case was 460 cGy. Figure 8 displays the locations of the measured data points in the neck phantom.

### M. MU verification

Current treatment planning systems may offer the option of calculating MUs, thus relating the dose distributions to the actual machine output. The methods by which the treatment planning systems relate dose distributions to machine output vary widely. For example, one commercial treatment planning system uses calibrated machine output obtained when the machine was originally commissioned as the starting point for MU calculations. In this method, the physicist enters the measured output at a specified reference point (usually a depth of 10‐cm depth) for a reference field size (usually 10cm×10cm), and for a reference distance (for example, 100‐cm SAD). Rather than normalizing the detector readings to the reading obtained under the reference conditions at the time of each set of measurements, calculations of the total scatter factor (TSF)^11^ were compared rather than the absolute number of MUs. The TSF is defined to be the output at the dose normalization point divided by the output at a 10‐cm depth for a 10cm×10cm field. Using the TSF for absolute dose determination removes the daily variation of the machine output from the measured data.

To test MU calculations, the TSF in the water phantom was measured at the normalization points for each of the ten water‐phantom test cases. TSFs were obtained by referencing the electrometer reading at the particular normalization point to the electrometer reading at a depth of 10 cm for a 10cm×10cm field at an SSD of 100 cm for each energy for 100 MU. To extract these TSFs from the commercial radiation treatment planning system, 100 MUs were prescribed for each test case, and the absolute dose was recorded and then divided by the dose for a 10cm×10cm collimator setting for each energy.

## RESULTS

### A. Water phantom test cases

**Figure 9:**
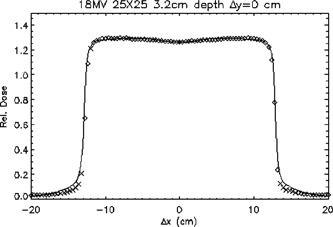
A transverse plane profile of an 18‐MV 25cm×25cm beam passing through the central axis at a depth of 3.2 cm. ◊; points meeting TG‐53 criteria, ×; points not meeting TG‐53 criteria. The continuous line represents ion chamber measurements.

Figure 9 shows the results of the comparison of the calculated and measured photon doses for an 18‐MV 25cm×25cm field, 100‐cm SSD, at a depth of 3.2 cm in a transverse plane. In this comparison, the computed tails of the dose profile are much flatter than the measured tails. The TG‐53 tolerance of ±2% for the outer region is not met for the points at ±13.6cm from the central axis. The TG‐53 tolerance of ±2% for the inner region is also not met for the point at −12.4 cm from the central axis.

**Figure 10:**
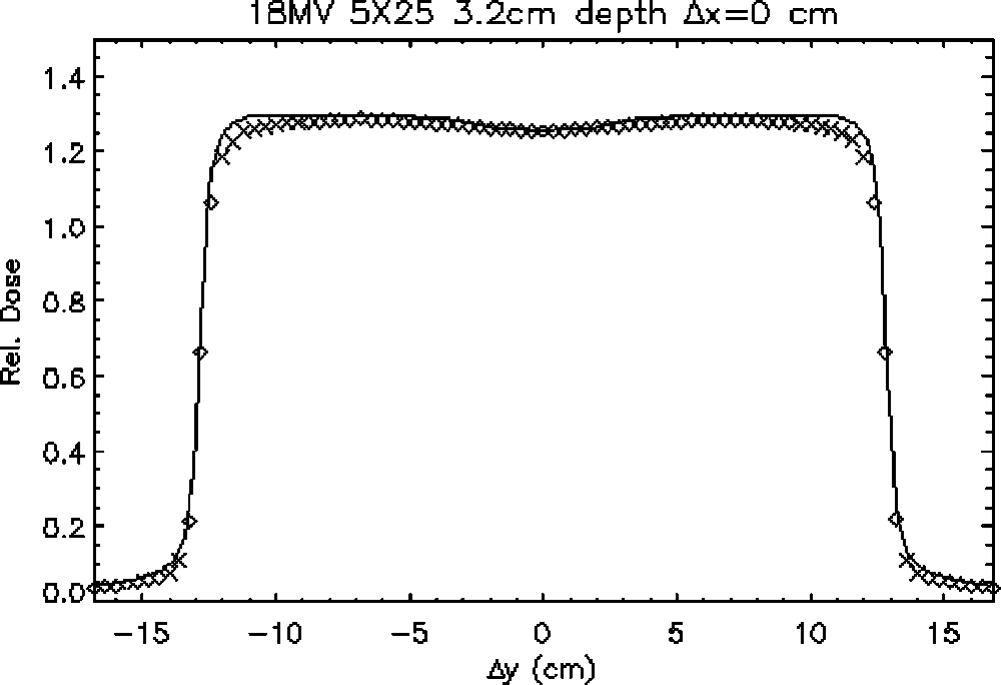
A sagittal plane profile of an 18‐MV 5cm×25cm beam passing through the central axis at a depth of 3.2 cm. ◊; points meeting TG‐53 criteria, ×; points not meeting TG‐53 criteria. The continuous line represents ion chamber measurements.

Figure 10 illustrates the plot of a profile for an 18‐MV 5cm×25cm field, 100‐cm SSD, at a depth of 3.2 cm in a sagittal plane. The calculated doses in the inner region away from the central axis are underestimated because of the way the radial dependence of the incident photon fluence was modeled.

**Figure 11:**
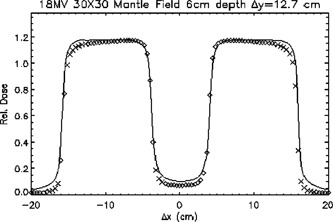
A transverse plane profile of an 18‐MV 30cm×30cm mantle field passing through the point at (0.0 cm, 12.7 cm) at a depth of 6 cm. ◊; points meeting TG‐53 criteria, ×; points not meeting TG‐53 criteria. The continuous line represents ion chamber measurements.

Figure 11 is a plot of an 18‐MV 30cm×30cm mantle field profile passing through the off‐axis point (0 cm, 12.7 cm) at a depth of 6 cm in the *x* direction. Discrepancies between calculation and measurement were observed in two regions. First, the calculated doses outside the field (in both the beam penumbra and under the block) underestimated the measured dose with dose differences beyond the TG‐53 criteria. Second, doses to the shoulders were also underestimated.

**Figure 12:**
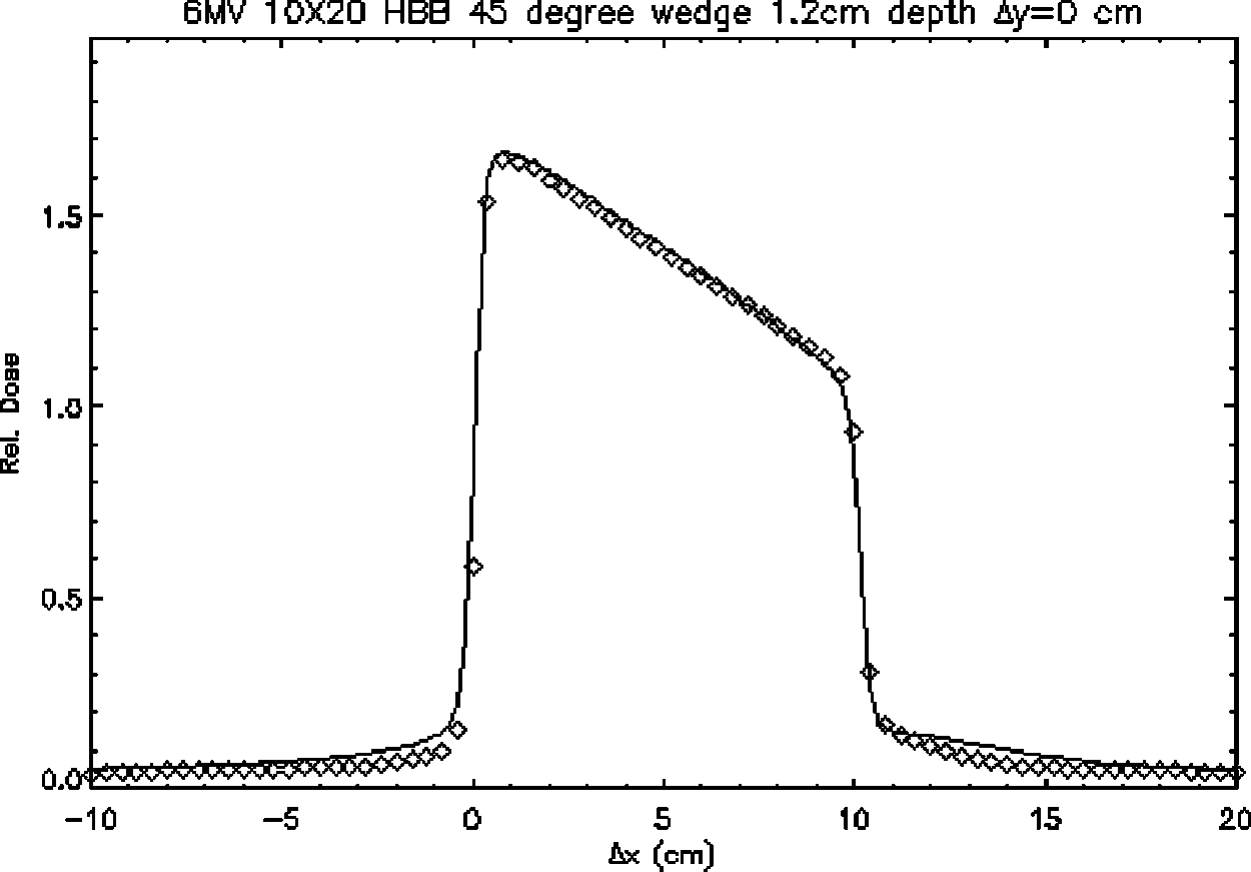
A transverse plane profile of a 6‐MV beam with a 45° wedge of length 20 cm with *x* collimators set asymmetrically at 0.0 cm and 10.0 cm passing through the central axis at a depth of 1.2 cm. ◊; points meeting TG‐53 criteria, ×; points meeting TG‐53 criteria. The continuous line represents ion chamber measurements.

Figure 12 illustrates a dose profile for a 6‐MV field, asymmetrically collimated to 10cm×20cm field, at a depth of 1.2 cm with a 45° wedge inserted in the *x* direction with the toe pointing toward gantry left. One of the collimator jaws is placed at the central axis of the beam. The TG‐53 criteria are normally specified as a percent of the central ray normalization dose. However, doses in this test case could not be normalized to a point on the central axis of the beam because the central axis lay in a region of high‐dose gradient. TG‐53 criteria were extended to this test case by establishing a normalization point in the approximate center of the radiation field. The depth of the profile was 1.2 cm and the profile passed through the central axis in a transverse plane. On the right‐hand side, doses in the tails were underestimated more towards the edge of the field.

**Figure 13:**
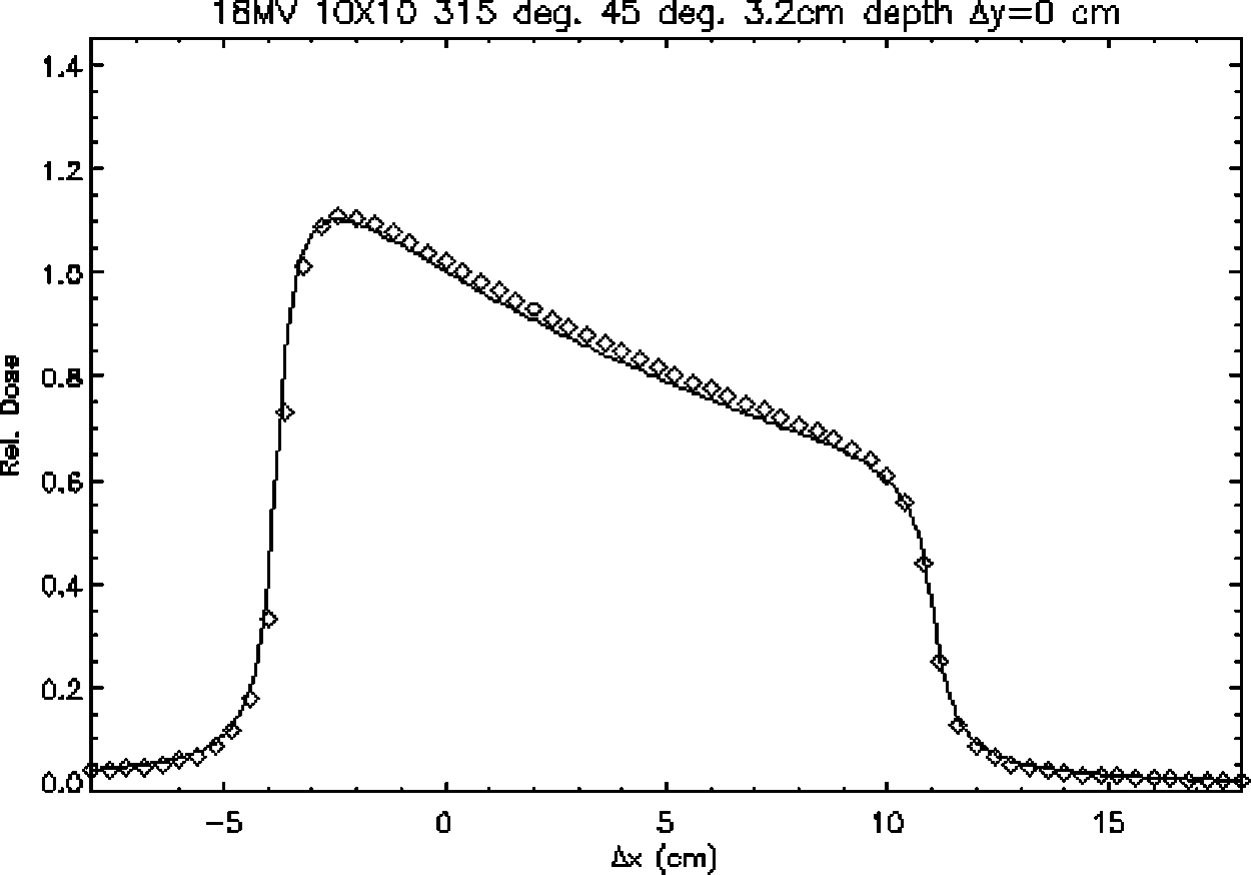
A transverse plane profile of an 18‐MV 10cm×10cm beam incident at an angle of 45° with a 45° wedge passing through the central axis at a depth of 3.2 cm. ◊; points meeting TG‐53 criteria, ×; points not meeting TG‐53 criteria. The continuous line represents ion chamber measurements.

Figure 13 is a plot of a profile of an 18‐MV 10cm×10cm beam incident at an angle of 45° with a 45° wedge measured at a depth of 3.2 cm passing through the central axis in a transverse plane. As with the beam shown in Fig. 12, doses in this test case were not normalized to a point on the central axis of the beam. However, the TG‐53 criteria were extended to this test case by establishing a normalization point in a region of high‐ and low‐dose gradient. All calculated profiles then agreed well with the measured data.

**Figure 14:**
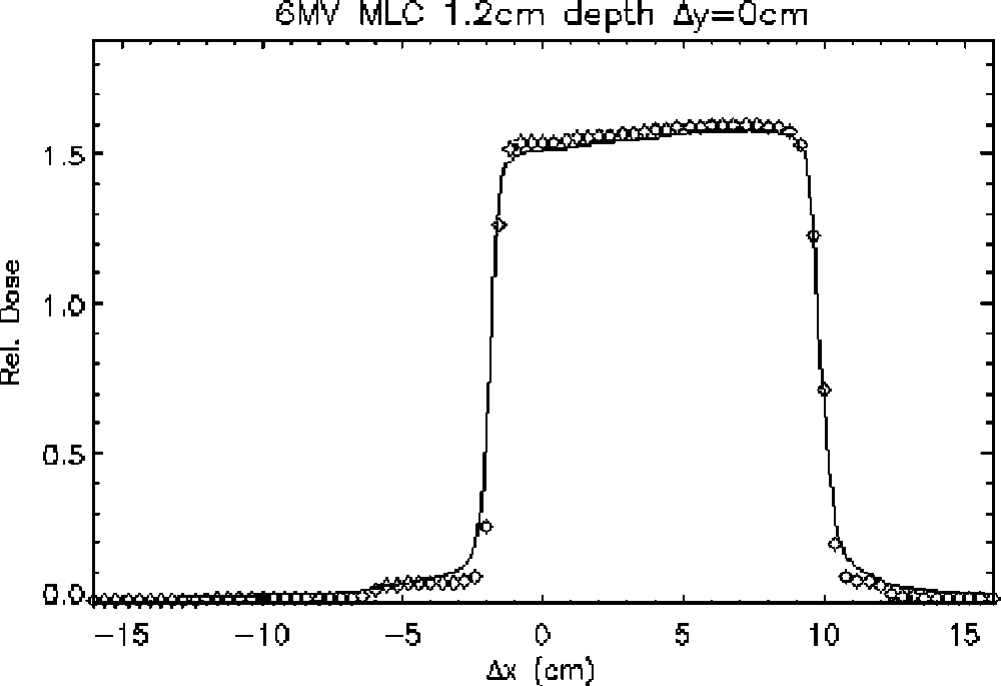
A transverse plane profile of a 6‐MV MLC‐shaped beam passing through the central axis at a depth of 1.2 cm. ◊; points meeting TG‐53 criteria, ×; points not meeting TG‐53 criteria. The continuous line represents ion chamber measurements.

Figure 14 is a plot of a 6‐MV MLC‐shaped field at a depth of 1.2 cm passing through the central axis in a transverse plane. This profile was in the buildup region. The treatment planning system matched the shape of the measurements fairly well. It should be noted, however, that the TG‐53 tolerances are high in the buildup region, namely ±20%.

Comparing calculations to measurements for all points in this study, we found the treatment planning system calculated photon doses to within the AAPM TG‐53 criteria for 99% of points in the buildup region, 90% of points in the inner region, 88% of points in the outer region, and 93% of points in the penumbra.

Table II summarizes the results of the monitor unit testing process. The numbers in the cells are the total scatter factors for each test situation. A noteworthy trend is seen in the table. Specifically, when modifiers or blocks were applied to the beam, the treatment planning system consistently underestimated the total scatter factor. The discrepancies in monitor units for the 18‐MV 5cm×5cm and 18‐MV 25cm×25cm beams also did not meet the TG‐53 criterion of ±0.5%. However, these criteria do not include the errors in determining the absolute dose under standard calibration conditions in their tolerance figures for the absolute dose at the normalization point. The criteria also do not provide for errors in determining the total scatter factor in their estimate for acceptable agreement. In addition, the errors in monitor units for rectangular fields exceeded the TG‐53 tolerance of ±0.5%. The error in monitor units for the mantle field also did not meet the TG‐53 criterion for blocked fields of ±1%. The error in monitor units for the oblique incidence field exceeded the TG‐53 criterion for external surface variations of ±0.5%. The error in monitor units for the last test case that exceeded the TG‐53 criteria was the oblique incidence with a wedge. Here, the TG‐53 criterion for wedges of ±2% was used because an explicit criterion for an obliquely incident field with a wedge does not exist.

**Table II:** Calculated and measured total scatter factors for the water phantom test cases.

		Measured	Pinnacle	% Error	Met Criteria
Test 1	6‐MV 5cm×5cm	0.895	0.898	0.50	Yes
	6‐MV 25cm×25cm	1.131	1.127	−0.39	Yes
	18‐MV 5cm×5cm	0.924	0.930	0.70	No
	18‐MV 25cm×25cm	1.077	1.071	−0.53	No
Test 2	6‐MV 10cm×10cm at 125 cm SSD^a^	0.655	0.657	0.27	Yes
	6‐MV 25cm×25cm at 125 cm SSD	0.744	0.737	−0.90	Yes
	18‐MV 10cm×10cm at 125 cm SSD	0.656	0.653	−0.40	Yes
	18‐MV 25cm×25cm at 125 cm SSD	0.710	0.709	−0.14	Yes
Test 3	6‐MV 25cm×5cm	0.955	0.963	0.83	No
	6‐MV 5cm×25cm	0.975	0.966	−0.96	No
	18‐MV 25cm×5cm	0.963	0.966	0.34	Yes
	18‐MV 5cm×25cm	0.985	0.977	0.81	No
Test 4	6‐MV 6cm×6cm 45° wedge	0.461	0.462	0.18	Yes
	6‐MV 20cm×20cm 45° wedge	0.560	0.557	−0.57	Yes
	18‐MV 6cm×6cm 45° wedge	0.492	0.493	−0.06	Yes
	18‐MV 20cm×20cm 45° wedge	0.571	0.568	−0.45	Yes
	6‐MV 6cm×6cm 60° wedge	0.385	0.386	0.12	Yes
	6‐MV 15cm×15cm 60° wedge	0.451	0.447	−0.98	Yes
	18‐MV 6cm×6cm 60° wedge	0.412	0.412	−0.11	Yes
	18‐MV 15cm×15cm 60° wedge	0.465	0.462	−0.76	Yes
Tset 5	6‐MV 30cm×30cm mantle	1.112	1.089	−2.12	No
	18‐MV 30cm×30cm mantle	1.073	1.050	−2.10	No
Test 6	6‐MV 10cm×10cm at 90 cm SSD	1.219	1.218	−0.12	Yes
	18‐MV 10cm×10cm at 80 cm SSD	1.519	1.511	−0.54	Yes
Test 7	6‐MV 10cm×10cm 330° obliquity	1.213	1.190	−1.84	No
	6‐MV 10cm×10cm 305° obliquity	1.266	1.240	−2.02	No
	18‐MV 10cm×10cm 330° obliquity	1.166	1.156	−0.87	No
	18‐MV 10cm×10cm 305° obliquity	1.220	1.207	−1.05	No
Test 8	6‐MV 10cm×20cm 45° wedge	0.413	0.410	−0.68	Yes
	18‐MV 10cm×20cm 45° wedge	0.441	0.436	1.10	Yes
Test 9	6‐MV 10cm×10cm 315° angle 45° wedge	0.726	0.708	−2.37	No
	18‐MV 10cm×10cm 315° angle 45° wedge	0.745	0.732	−1.70	Yes
Test 10	6‐MV MLC^b^	0.997	0.997	0.03	Yes

a SSD is defined as the source‐to‐surface distance.

b MLC is defined as a multileaf collimator.

### B. Heterogeneous phantom test cases

Table III shows the measured and calculated doses in the anthropomorphic lung phantom test case. The last column in the table, the standard error of the mean, demonstrates the precision of the TLD measurements. It is interesting to note that doses to all of the points except one were underestimated by the treatment planning system. The calculated dose to Point 7 deviated the most from the measurements. However, this point was in the penumbra of the beam. Of the points within the beam, the dose at Point 5 deviated the most from the measurements. This could be due to the underestimation of scatter from nearby bone or to the fact that this point had the highest standard error of the mean. In the presence of heterogeneities with significantly different average atomic numbers, such as lung and bone, electron transport should be dealt with explicitly.^12^ If we apply the TG‐53 criteria, all dose discrepancies were within the specified limits of ±7% in the outer region, ±7% in the inner region, or 7 mm in the penumbra. A previous study verifying calculations from this commercial radiation therapy treatment planning system against Monte Carlo‐generated dose distributions on treatment plans found all calculations were within ±2.6% of the Monte Carlo‐generated data.^13^ In fact, a previous study conducted within this institution comparing treatment plans for large‐breasted patients and measurements obtained by thermoluminescent dosimetry (TLD) in an anthropomorphic phantom found the calculated doses to all points were within ±3% of the measured doses.^14^

**Table III:** Calculated and measured point doses for the anthropomorphic lung phantom test case.

Point No.	TLD^a^ (Gy)	Pinnacle (Gy)	% Error	Standard error of mean (Gy)
P2	5.27	5.19	−1.52	0.0167
P3	4.33	4.27	−1.39	0.0100
P4	5.08	5.01	−1.38	0.0145
P5	4.78	4.65	−2.72	0.0473
P6	4.31	4.38	1.62	0.0219
P7	3.64	3.36	−7.69	0.0067

a TLD is defined as measurements obtained via thermoluminescent dosimetry.

Table IV displays the measured and calculated doses for the anthropomorphic neck phantom test case. Doses to all test points were overestimated by the treatment planning system except for Point 5, which was located in the spinal cord. This dose underestimation is consistent with the previous test case point that was close to bone. Point 3, which lies on the left edge of the spinal cord, exhibited the largest dose discrepancy. However, the standard error of the mean for this measurement was the largest of all the measured data points. Again, there was good agreement between the calculated dose values and the measured dose values.

**Table IV:** Calculated and measured point doses for the anthropomorphic neck phantom test case.

Point No.	TLD^a^ (Gy)	Pinnacle (Gy)	% Error	Standard error of mean (Gy)
P2	3.54	3.60	1.69	0.0321
P3	4.36	4.49	2.98	0.0926
P4	4.02	4.08	1.49	0.0186
P5	3.86	3.81	−1.30	0.0167

a TLD is defined as measurements obtained via thermoluminescent dosimetry.

## DISCUSSION

The primary cause for discrepancies between calculations and measurements were deficiencies in the beam model. For small, square open fields (5cm×5cm), the calculated shoulders and tails underestimated the measured data. The underestimation resulted because parameters that described the finite source size and stray scatter from the head had to be modified so that monitor unit calculations would closely match clinical data, thus compromising the accuracy of calculations in the shoulders and tails.^7^ For large, square open fields (25cm×25cm), calculations overestimated measurements in the tails, because the parameter that described stray scatter from the head was also modified so that monitor unit calculations would closely match clinical data. Inaccuracies in modeling scatter were also evident in the effect of modifiers or blocks on the accuracy of monitor unit calculations. A possible remedy to the extra focal radiation problem is to use a dual‐source photon beam model.^15^

Calculated profiles along the long axis of elongated fields (5cm×25cm or 25cm×5cm) underestimated measurements in the shoulder region, while calculated profiles along the short axis overestimated measurements. These inaccuracies occurred because of the manner in which the radial distribution of the in‐air fluence was modeled. Specifically, the incident photon fluence was assumed to increase linearly with the distance from the central axis until a certain boundary, beyond which the fluence was assumed to be flat. Thus, two parameters specified the incident fluence: a cone angle, which described the rate of increase in the fluence as the off‐axis distance increased; and a cone radius, which described the point at which the fluence profile became flat.^16^ In the treatment planning system, all rectangular fields were modeled with cone angles and cone radii for the equivalent square‐field size. In commissioning this beam, the cone radius was taken to be field‐size dependent to match calculation with measurement. A more realistic beam model, however, would have a cone radius independent of the field size. For the 5cm×25cm field, the equivalent square is 8.3cm×8.3cm. The cone radius that should have been used for this field was the one for a 25cm×25cm field. Similarly, the cone radius that should have been used for the profiles acquired in the *x* direction for this setup was the cone radius for a 5cm×5cm field. Consequently, the cone radius of 7 cm, which would have been appropriate for an 8.3cm×8.3cm field, resulted in a cutoff of the fluence increase at too small a radius for the 25‐cm width of the 5cm×25cm field.

Calculations in blocked fields underestimated measurements both in the tails and in the shoulders, as seen in Fig. 11. The underestimation of dose in the tails may be due to inaccurate modeling of the attenuation and scatter from the block, while the underestimation of dose in the shoulders may also be due to inaccurate modeling of the fluence profile within the field.

Calculations in wedged fields underestimated measurements in the tails on the side of the heel of the wedge and in the shoulder near the toe of the wedge. These discrepancies were due to the symmetric nature of the parameters that were radially dependent such as the Gaussian height parameter, which accounts for more head scatter and modifies the calculated dose in the both tails and the cone angle, which accounts for the profile of the in‐air fluence. In the case of a wedge, the relative dose profile is not radially symmetric, resulting in a compromise when selecting the cone radius and cone angle. Moreover, the beam model does not directly account for wedge‐generated scatter. One remedy to this situation is to include the wedge in the calculation volume, as in the extended phantom model.^17^ The beam model also does not address differential hardening from the wedge. Consequently, calculated depth doses tend to underestimate measurements at deeper depths and overestimate measurements at shallower depths. Calculated doses outside the field yet under MLC leaves were underestimated because interleaf leakage was not modeled.

Ion chamber measurements indicate that doses to most of the calculated points are acceptable according to the TG‐53 criteria. The sources of the deviations from the criteria were identified. TLD measurements indicated that the treatment planning system accurately predicted doses in heterogeneous media to within ±3%. However, the generally stated goal of dose delivery accuracy to within 5% was not met in all situations with this beam model. Clinically, the greatest difficulty is posed by rectangular fields, where the inner region of the beam was underestimated by as much as 9.75% in some cases. Also, the monitor unit calculations for the oblique incidence cases show deviations around 2.4%, which is considered borderline acceptable in a clinical context.

To compare calculated and measured doses, the TG‐53 report divided the beam into several regions, the buildup region, the inner region, the penumbra, and the outer region. The tolerances for the buildup region range from ±20% for open fields at standard SSD to ±50% for wedged fields. The present study found only six points out of 4138 points exceeding the TG‐53 criteria for the buildup region. All six points occurred in the MLC‐shaped field test case. The errors that were typically encountered were less than ±20%. According to the TG‐53 report, dose acceptability criteria were based on the collective expectations of the members of the task group and were not to be used as goals or requirements for any particular situation. The present work indicated that the TG‐53 dose acceptability criteria for the buildup region are too forgiving and may require adjustment. Furthermore, the buildup region might be construed as a region of a high‐dose gradient and a distance criterion might be used rather than a dose criterion. The criteria cited by Venselaar *et al.* ^18^ of 10–15% or 2–3 mm might be more appropriate here.

A shortcoming of the TG‐53 report may be in how the various regions are defined. For example, the TG‐53 report defines the penumbra as the region from 0.5 cm inside to 0.5 cm outside the beam/modifier edge. However, this definition does not allow for broadening of the penumbra with depth. This leads to a definition of the penumbra that may not encompass the entire high‐dose‐gradient portion of the beam. For example, as was seen in Fig. 9, several points in the calculated 18‐MV 25cm×25cm beam failed to meet the TG‐53 tolerance of ±2% for the outer and inner regions. According to the TG‐53 definitions, these points should lie in either the outer or inner regions, but such assignment is questionable because the points are located in a region of steep dose gradient. Consequently, the penumbra of the beam might be better defined by a criterion based on slope. For example, the penumbra for a square, open field could be defined to be in the region where the magnitude of the slope is ≥3% per mm, as was suggested by Venselaar *et al* ^18^ Such a definition must also ensure that the slope search occurs in a region containing the 50% isodose level to prevent the definition from satisfying the slope limits in the center of the field or other points that do not include the edge of a beam/modifier edge.

Lastly, the TG‐53 criteria do not specify tolerances for regions of electronic disequilibrium in heterogeneous media. Goals in the buildup region for heterogeneous media also need to be defined; otherwise one would be unable to judge whether the algorithm is predicting dose acceptably in these situations.

Although this study was performed on a software version (Version 4.2) that has since been superseded, the same analysis can be performed on newer versions of the software as well as on other radiation treatment planning software.

## CONCLUSIONS

We have generated a measured data set for verifying photon dose calculations. In contrast to previous data sets, this set includes measured TSFs, and measurements in an anthropomorphic phantom. The effects of oblique incidence with a wedged field, asymmetric collimation with a wedged field, mantle‐field irradiation, and use of an MLC were also studied.

This data set was designed so that it could be used for general verification of photon dose‐calculation algorithms. Indeed, the first test case can be used to generate the beam model, and the subsequent test cases can be used to validate the dose‐calculation accuracy under various situations. The data set is available on request for anyone wishing to verify their beam model. Further information on obtaining the data set can be obtained via the Radiological Physics Center web site (http://rpc.mdanderson.org).

## ACKNOWLEDGMENTS

This work was supported in part by a Sponsored Research Agreement with ADAC Laboratories and NIH Grant No. CA‐10953 to the Radiological Physics Center.
